# The Performance of Flexible Tip Bougie™ in Intubating Simulated Difficult Airway Model

**DOI:** 10.3389/fmed.2021.677626

**Published:** 2021-05-07

**Authors:** Nurfadilah Mahli, Jaafar Md Zain, Siti Nidzwani Mohamad Mahdi, Yeoh Chih Nie, Liu Chian Yong, Ahmad Fairuz Abdul Shokri, Muhammad Maaya

**Affiliations:** Department of Anaesthesiology & Intensive Care, Faculty of Medicine, Universiti Kebangsaan Malaysia Medical Centre, Kuala Lumpur, Malaysia

**Keywords:** anesthesiology trainee, bougie, difficult intubation, direct laryngoscopy, flexible tip bougie, simulation

## Abstract

This prospective, randomized, cross-over study compared the performance of the novel Flexible Tip Bougie™ (FTB) with a conventional bougie as an intubation aid in a simulated difficult airway manikin model among anaesthesiology trainees with regards of first pass success rate, time to intubation, number of attempts and ease of use. Sixty-two anesthesiology trainees, novice to the usage of FTB, participated in this study. Following a video demonstration, each participant performed endotracheal intubation on a manikin standardized to a difficult airway view. Each participant performed direct laryngoscopy and intubated the manikin using a conventional bougie and FTB, at least 1 day in between devices, in a randomized order. The first pass success rate was significantly higher with FTB (98.4%) compared to conventional bougie (85.5%), *p* = 0.008. The median time to intubation was significantly faster when using FTB, median = 32.0 s [Interquartile range (IQR): 23.8–41.3 s] compared to when using conventional bougie, median = 41.5 s (IQR: 31.8–69.5 s), *p* < 0.001. The FTB required significantly less intubation attempts compared to conventional bougie, *p* = 0.024. The overall ease of use, scored on a Likert scale from 1 to 5, was significantly higher in the FTB (4.26 ± 0.53) compared to the conventional bougie (3.19 ± 0.83), *p* < 0.001. This simulated difficult airway manikin study finding suggested that FTB is a useful adjunct for difficult airway intubation. The FTB offered a higher first pass success rate with a faster time to intubation and less required attempts.

## Introduction

Tracheal intubation is an essential skill that must be acquired by an anesthesiologist. While the incidence of difficult intubations was stated to be 6–11%, a failed intubation which is a more serious problem, varies in different settings (1). It can be ≈1 in 2,000 for elective cases, ≈1 in 300 during rapid sequence induction for the obstetric cases, and about ≈1 in 50–100 in the emergency department, intensive care unit and pre-hospital setting (2). Various strategies currently employed to manage difficult intubation ranges from simple adjuncts, such as the bougie, to the more sophisticated devices, like the videolaryngoscope. According to the Malaysia National Audit on Anesthetic Airway Management (2015), bougie was the second most preferred (24.8%) airway adjunct of choice in a difficult airway event, following the videolaryngoscope (44.6%) (3).

**Figure 1:**
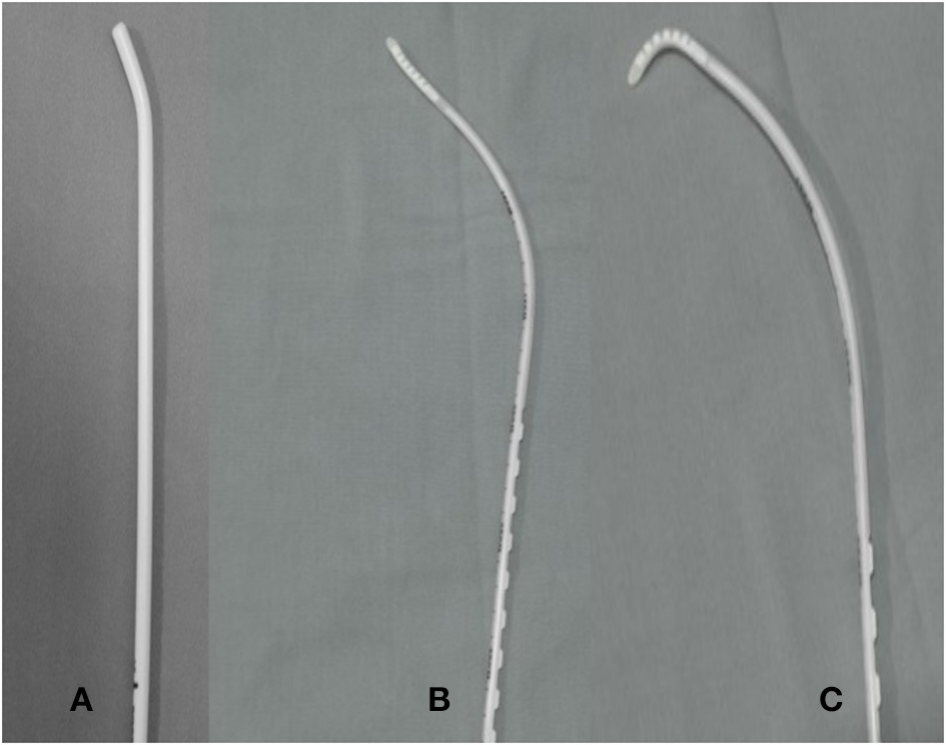
The conventional bougie (Portex®, coude tip, 15 F) **(A)** shown in comparison to The Flexible tip bougie™ in neutral **(B)** and in anterior flexion **(C)**.

The use of a bougie, a simpler and cheaper device compared to the videolaryngoscope, first described by Macintosh in 1949, may increase the first pass success (FPS) of an endotracheal tube (ETT) placement by 78–100%, especially in poor laryngeal view (4, 5). The bougie (Figure 1A) is commonly about 15 F in diameter and 70 cm in length with the tip angled at 30° to facilitate its navigation toward an anteriorly located larynx (6). Successful endotracheal intubation with the bougie involves its insertion into the trachea, followed by sliding the ETT via the Seldinger technique (7). Common difficulties in bougie-assisted intubation are the inability to insert the bougie past the hypopharynx (14.8%), the inability to pass the ETT over the bougie (6.8%) and esophageal intubation (4.5%) (8). The bougie can also bend in the hypopharynx during the attempt to direct it anteriorly.

The Flexible Tip Bougie™ (FTB) by Construct Medical (Australia) (Figure 1B) has a mid-shaft slider which can be used to angle the tip anteriorly (Figure 1C) and posteriorly, thereby facilitating placement through the vocal cords. This flexibility may facilitate maneuvering the bougie past the hypopharynx as compared to the conventional bougie that has the tip fixed at 30°. The smooth silicon tip of the bougie also minimizes the risk of tracheal trauma during intubation, which is an advantage when dealing with a larynx with a very anterior view.

In this study, we evaluated the performance of the FTB in a simulated difficult airway model in comparison to a conventional bougie when used by anesthesiology trainees in terms of FPS, time needed for intubation and number of attempts for both types of bougie.

## Materials and Methods

This prospective, randomized, cross-over simulation study was approved by the Research Committee of Department of Anaesthesiology & Intensive Care, Universiti Kebangsaan Malaysia Medical Center (UKMMC) and the Medical Research & Ethics Committee, UKMMC (code no: FF-2019-356). Following approval and written consent, 62 anesthesiology trainees in UKMMC participated in this study. All participants had ample experience using the conventional bougie. Any participant who had prior experience with FTB were excluded from this study. Data on the participants' gender, years of clinical experience and the year of postgraduate anesthesia training were collected.

A video demonstration on intubation with FTB, slightly <2 min duration, produced by the manufacturer was shown to the participants who were then allowed to familiarize themselves in maneuvering the tip of bougie before proceeding to performance testing. There was no practice intubation with the bougie prior to the performance testing. The airway simulation model used was the Laerdal® Airway Management Trainer, which was simulated to a “Difficult view” (9). The “Difficult view” was achieved by application of a hard cervical collar on the manikin and verified by the same investigator to be a partial glottis opening of ~20%. This mimics a Grade 2b Cormack-Lehane glottis view which is similar to an anterior larynx seen during laryngoscopy.

All participants were provided with standardized equipment, which were a 7.5 mm ETT, a Macintosh laryngoscope blade size 4, a bag valve mask device and a 10 ml syringe. Each participant performed direct laryngoscopy and intubated the manikin using a conventional bougie (Portex® Single Use Bougie coude tip, 15 F) and FTB in a randomized order determined by chance picking of binary numbers. Following intubation, the ETT was ventilated with the bag valve mask device and successful intubation was confirmed by lung inflation. At least a period of 1 day was observed before the participants attempted intubation using the other type of bougie. Any participant who did not return for the second placement test were considered as dropouts.

The primary outcome was the FPS rate, which was defined as successful placement of the ETT in the trachea on the participant's first attempt at laryngoscopy. The attempt was considered unsuccessful if there was no lung inflation or removal of the ETT with the bougie from the manikin. A failed intubation was defined as either failed three attempts of lung ventilation through the ETT or after 3 min of procedure, whichever occurred earlier. Any occurrence of esophageal intubation, which was insertion of the ETT into the esophagus resulting in stomach inflation were also recorded.

The secondary outcome was the time required for a successful tracheal intubation. The time to successfully intubate, measured to one decimal point using a stopwatch, was taken from the moment when the laryngoscope blade enters the mouth and ends with lung inflation with bag mask ventilation. After completion of intubation with both types of bougie, the participants were asked to complete a survey to assess the overall ease of intubation with both techniques in this simulated airway. The trainees were asked to score according to a five-point Likert scale of 1 (very difficult) to 5 (very easy) on the ease of passing both types of bougies through the hypopharynx, sliding the ETT over the bougie and maneuvering the flexible tip slider.

From a previous study, the FPS rate in utilizing conventional bougie in a simulated difficult airway was 75% (10). In this prospective crossover study, by setting the α = 0.05 with the β = 0.80 and using Statulator, the sample size required was 62 participants to detect a 20% difference of FPS between bougies types (11).

All statistical analyses were performed with the statistical package for social science (SPPS) statistical software (ver26, NY: IBM Corp). The data was analyzed in three phases. Firstly, descriptive statistics were computed for all variables of interest. The normal distribution of data was tested using the Kolmogorov-Smirnov test. McNemar's test was used to determine whether the FPS rate was different between the two types of bougies. Due to distribution characteristics, non-parametric statistics were used, namely Wilcoxon Signed-rank test to determine the difference between times to intubation. Fisher exact test was used to analyze the association of attempts when using both bougies. The ease of use perceived by the participants was analyzed with paired *t*-test. Data were presented as median with interquartile range (IQR), number with percentage (%), or mean with standard deviation, as appropriate.

## Results

A total of 62 anesthesiology trainees in UKMMC participated in this study with a median of 6 years (IQR: 5–7) clinical experience in anesthesia (Table 1). There was no dropout.

**Table 1:** Demographic data, clinical experience, and year of postgraduate anesthesia training.

**Parameters (*n* = 62)**	
**Gender**	
Female	37 (59.7%)
Male	25 (40.3%)
Anesthesia clinical experiences (years)	6 (IQR: 5–7)
**Year of postgraduate anesthesia training**	
Year 1	9 (14.6%)
Year 2	15 (24.2%)
Year 3	20 (32.2%)
Year 4 and above	18 (29.0%)

*Data presented as number (percentage), or median (interquartile range), as appropriate.*

The FPS rate when using FTB and conventional bogie was 98.4 and 85.5%, respectively. A total of eight participants who did not achieve FPS when using conventional bougie were successful on the first intubation attempt when FTB was used. An exact McNemar test determined that the change in the proportion of FPS was statistically significant (*p* = 0.008) as seen in Table 2.

**Table 2:** The change of first pass success (FPS) when using Flexible Tip Bougie™ Values expressed in number.

		**Flexible tip bougie**™		
		FPS^*^	**Not FPS**	**Total**
Conventional bougie	FPS	53	0	53
	Not FPS	8	1	9
	Total	61	1	(*n* = 62)

* *McNemar, p = 0.008.*

Table 3 showed the comparison of FPS, intubation attempts, time to intubation, failed intubation and esophageal intubation. One participant (1.6%) was considered to have failed intubation as a duration of 181 s was taken with the conventional bougie. This participant took three attempts with the conventional bougie but was able to achieve FPS with FTB. Five participants (8.1%) intubated the esophagus when using the conventional bougie, whilst none occurred with the FTB. This finding was not statistically significant.

**Table 3:** Comparison of first pass success (FPS), intubation attempts, time to intubation, failed intubation, esophageal intubation, and ease of use for each type of bougie.

	**Flexible tip bougie™**	**Conventional bougie**	***p*-value**
No. of intubation attempts			0.024
1 (FPS)	61 (98.4%)	53 (85.5%)	0.008
2	1 (1.6%)	6 (9.7%)	
3	0 (0.0%)	3 (4.8%)	
Time to intubation, sec	32.0 (23.8–41.3)	41.5 (31.8–69.5)	<0.001
Failed intubation	0 (0.0%)	1 (1.6%)	NS
Esophageal intubation	0 (0.0%)	5 (8.1%)	NS
Ease of use	4.26 (± 0.53)	3.19 (± 0.83)	<0.001
Ease of bougie passing hypopharynx	4.47 (± 0.59)	3.48 (± 1.04)	<0.001
Ease of ETT passing over bougie	4.56 (± 0.74)	4.08 (± 0.91)	0.002

*Data presented as median (interquartile range) or number (percentage) as appropriate.*

*p < 0.05 is significant, NS, Not statistically significant.*

**Figure 2:**
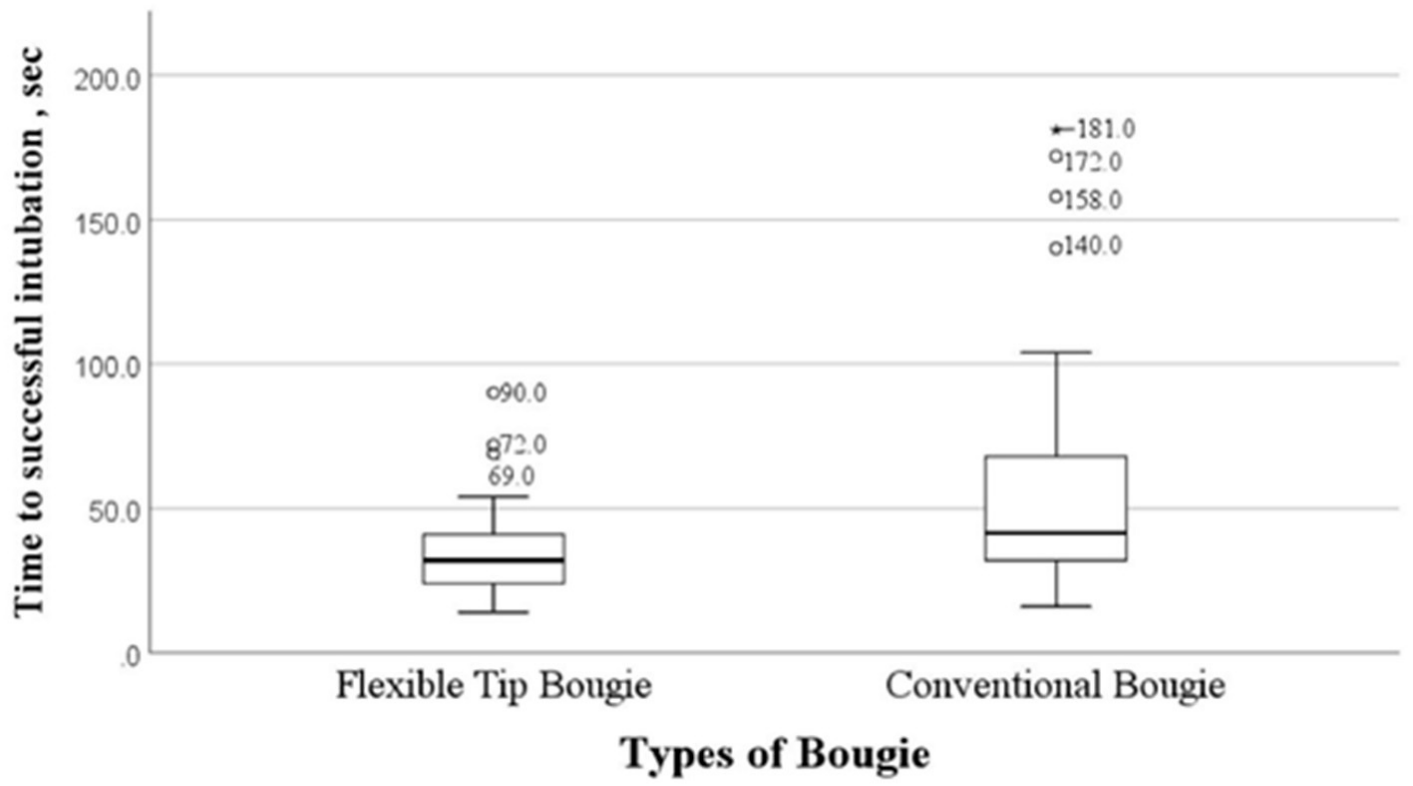
Time to successful intubation (sec) of conventional bougie and Flexible Tip Bougie™. Values expressed in seconds (sec).

Figure 2 showed the time to successful intubation for both types of bougies. Four participants required more than 120 s to intubate when using conventional bougie due to the difficulty in laryngoscopy to achieve satisfactory glottic view prior to attempting intubation followed by further difficulty in manipulating the conventional bougie through the hypopharynx. These four participants had a range of 3 to 9 years of experience in anesthesia.

Reliability test was performed on the data of the ease of each type of bougie usage and a Cronbach's alpha of more than 0.7 was accepted as a reliable cut-off point. Overall, the participants perceived that FTB was easier to use with a mean score of 4.26 ± 0.53 compared to 3.19 ± 0.83 for the conventional bougie (*p* < 0.001) as seen in Table 3. The FTB was deemed easier to pass through the hypopharynx with a mean score of 4.47 ± 0.59 compared to the conventional bougie, which had a mean score of 3.48 ± 1.04. Passing of an ETT over the bougie was perceived as relatively easy in both the FTB and conventional bougie, with a mean score of 4.56 ± 0.74 and 4.08 ± 0.91, respectively. The participants perceived that it was easy to maneuver the slider of FTB, with a mean of 4.16 ± 0.81.

## Discussion

As the incidence of difficult and failed intubation varies, this study was conducted using a standardized simulated model (2). Despite the low technology level involved in our study, the manikin was prepared to provide the fidelity akin to difficult airway view of a real patient, thus avoiding the issue of patient safety (12). The FPS is often promoted as the goal of intubation because as the number of attempts increases, the incidence of adverse events such as aspiration, hypoxemia and esophageal intubation will likely increase considerably (13). Furthermore, the principles of securing the airway safely, accurately, and swiftly is of utmost importance for intubation, an aerosol-generating procedure, during the Covid-19 pandemic (14). Repeated intubation attempts would increase the exposure and infection risk to the anesthesiologists and other healthcare workers involved. Our study found a higher FPS rate when using the FTB as an intubation adjunct compared to the conventional bougie in a simulated difficult airway manikin. Similarly, Báczek found novice paramedics had a higher FPS rate with the FTB compared to the conventional bougie during cardiopulmonary resuscitation simulation (15). Additionally, in our cross-over study, the exact McNemar test found the change in the proportion of FPS was statistically significant in favor of the FTB.

We reported a statistically significant shorter time to a successful intubation by 9.1 s with lesser number of attempts required when the trainees used FTB compared to conventional bougie as an intubation aid. A recent simulation study with cervical immobilization compared the use of FTB and standard bougie also reported a faster duration of to a successful intubation with the FTB, 37 vs. 46 s (*p* < 0.001) (16). Another manikin study evaluated the usage of the two bougies for intubation during cardiopulmonary resuscitation and reported a statistically shorter intubation time (21.4 s) with the FTB compared to the gum elastic bougie (25.7 s), *p* < 0.001 (15). The shorter time to intubation suggested that the flexibility of the FTB produced better and faster steering than the conventional bougie to achieve a successful intubation.

The FTB was perceived as easier to be used compared to the conventional bougie amongst our trainees. Ruetzler et al. also reported that the FTB was easier to use in difficult intubation situations especially in scenarios that involve limited cervical movement (16). The curve of the FTB resembles the anatomical airway curvature, thus facilitating its insertion into the hypopharynx region. This curvature can further be manipulated to swivel using the slider tab which provides further anterior and posterior movement of the tip and along with rotation, producing a 360° rotation aiding intubation, as reported in a case series (17). The FTB also has a bright phosphorous coating on its tip to enhance the bougie tip visibility to the intubator.

The findings of this study should be considered with a certain limitation. The high success rate could be attributed to the study being done among anesthesiology trainees that have plenty of clinical experience in intubation despite their novice experience to the FTB. Even though the result should not be generalized to physicians of different levels of experience, this study had provided a promising ground that the skill of the FTB usage can be learnt rapidly.

## Conclusion

This simulated difficult airway manikin study finding suggested that FTB is a useful adjunct for difficult airway intubation. The FTB offered a higher FPS rate with a faster time to intubation and less required attempts.

## Data Availability Statement

The raw data supporting the conclusions of this article will be made available by the authors, without undue reservation.

## Author's Note

Difficult and/or failed intubation, both anticipated and unanticipated, can be challenging and may result in patients' mortality or morbidity if not managed accordingly. Amongst the many airway management devices, the bougie is a simple, cheap, and accessible airway which can be used to manage the difficult airway. A simulation study is ideal for assessing the performance of a new device as it avoids the issue of patient safety and produces repeatable standardized situations. This cross-over study compared the performance an existing device with a novel device which has integrated a feature to enable the tip of the bougie to be flexed more anteriorly or posteriorly. As difficult intubation is relatively uncommon, the intubator can benefit by having an airway adjunct which can be utilized properly without extensive training. The primary and secondary objectives selected to assess the performance of both the novel and conventional bougies reflected the real-life challenges presented when dealing with a difficult airway. First pass success, faster time to successfully intubate and fewer attempts results in lesser morbidity related to a difficult or failed intubation.

## Author Contributions

NM and MM: investigators, proposal writing & editing, data analysis, and manuscript writing & editing. NM: data collection. JM, SM, YC, LC, AS, and MM: manuscript critic. All authors contributed to the article and approved the submitted version.

## Conflict of Interest

The authors declare that the research was conducted in the absence of any commercial or financial relationships that could be construed as a potential conflict of interest.
